# Myosteatosis in type 1 diabetes: immunometabolic mechanisms, biomarkers, and therapeutic targets

**DOI:** 10.3389/fendo.2026.1900161

**Published:** 2026-09-07

**Authors:** Fatema Al-Rashed, Halemah AlSaeed, Kevin Patrick Fennelly, Fahd Al Mulla, Rasheed Ahmad

**Affiliations:** 1 Immunology and Microbiology Department, Dasman Diabetes Institute, Kuwait City, Kuwait; 2 Division of Intramural Research, National Heart, Lung, and Blood Institute, National Institutes of Health, Bethesda, MD, United States; 3 Translational Research Department, Dasman Diabetes Institute, Kuwait City, Kuwait

**Keywords:** AMPK, inflammation, mitochondrial dysfunction, myosteatosis, oxidative capacity, PPARδ, SIRT1, type 1 diabetes

## Abstract

Myosteatosis, defined as pathological lipid accumulation within and between skeletal muscle fibers, is increasingly recognized as a determinant of impaired muscle quality, metabolic inflexibility, and adverse clinical outcomes. Although well described in ageing, obesity, and cancer, its relevance to type 1 diabetes (T1D) remains underexplored. T1D is characterized by lifelong insulin deficiency, persistent autoimmune activation, and glycemic variability, conditions that profoundly disrupt cellular energy metabolism and substrate utilization in skeletal muscle, even in the absence of obesity or overt sarcopenia. This review integrates evidence from human imaging, metabolic phenotyping, immunological profiling, and multi-omics analyses to define myosteatosis as an immunometabolic phenotype in T1D. Central to this framework is dysregulation of the AMP-activated protein kinase (AMPK)-peroxisome proliferator-activated receptor (PPAR)-mitochondrial axis, which normally coordinates fatty-acid oxidation, mitochondrial biogenesis, and energy efficiency in skeletal muscle. In T1D, chronic immune activation and metabolic stress suppress AMPK and PPARδ signaling, impair PGC-1α–dependent mitochondrial function, and reduce oxidative capacity, promoting intramyocellular lipid accumulation despite preserved muscle mass. These defects are reinforced by persistent inflammatory signaling (IL-6, TNF-α, IL-1β; NF-κB, JNK, and NLRP3 pathways), accumulation of lipotoxic intermediates (ceramides and diacylglycerols), dysregulated myokine secretion (increased myostatin with reduced IL-15 and irisin), and infiltration of pro-inflammatory macrophages and CD8^+^ T cells. Mitochondrial stress, reflected by impaired phosphocreatine recovery, altered acylcarnitine profiles, increased oxidative damage, and reduced NAD^+^–SIRT1/3 activity, further consolidates immunometabolic dysfunction and lipid deposition. Collectively, this review positions myosteatosis as a clinically relevant and potentially modifiable consequence of immune-driven failure of cellular energy utilization in T1D. Because direct mechanistic data from T1D skeletal muscle remain scarce, the framework presented here is deliberately hypothesis-generating: it is assembled substantially by inference from type 2 diabetes (T2D), obesity and ageing models, and we map the resulting evidence gaps explicitly in order to define a research agenda rather than to assert a validated T1D-specific mechanism. Targeting the AMPK-PPAR-mitochondrial axis and its inflammatory and lipotoxic modifiers may enable earlier detection and mechanism-based interventions to preserve muscle metabolic resilience and functional capacity in autoimmune diabetes.

## Highlights

- Myosteatosis is a distinct immunometabolic phenotype in type 1 diabetes independent of obesity.
- Chronic autoimmune activation disrupts the AMPK-PPAR-mitochondrial axis, promoting intramyocellular lipid accumulation.
- Ceramide and diacylglycerol accumulation, NLRP3 inflammasome activation, and mitochondrial dysfunction drive myosteatosis in a self-perpetuating cycle.
- Circulating acylcarnitines, ceramides, oxidized lipids, and mitochondrial DNA offer translatable biomarkers for early detection and disease stratification.
- Exercise, nutritional interventions, and pharmacological modulation of the AMPK-PPAR-NAD^+^ axis represent mechanistically informed therapeutic strategies.

## 1 Introduction

Myosteatosis, defined as the pathological accumulation of lipid within and surrounding skeletal muscle fibers, has emerged as a distinct indicator of impaired muscle quality and metabolic dysfunction (1, 2). Muscle quality here denotes the functional and metabolic competence of muscle per unit mass, as opposed to muscle bulk: force output and oxidative capacity fall as contractile tissue is displaced by lipid, even where cross-sectional area is preserved. While historically associated with obesity and ageing, myosteatosis is now increasingly recognized in chronic inflammatory and metabolic disorders, including T1D, where it reflects the convergence of immune dysregulation, altered substrate metabolism, and mitochondrial stress (3, 4). In T1D, lifelong dependence on exogenous insulin and fluctuating glycemic control may fail to fully recapitulate physiological insulin signaling in skeletal muscle, predisposing to ectopic lipid deposition, oxidative stress, and inflammatory activation that compromise muscle metabolic integrity even in the absence of overt atrophy (4–7). Beyond its role in locomotion and glucose disposal, skeletal muscle functions as a dynamic endocrine and immunoregulatory organ. It accounts for the majority of postprandial glucose uptake and secretes bioactive myokines, including interleukin-6 (IL-6), interleukin-15 (IL-15), myostatin, and irisin, which collectively regulate immune-cell activity, mitochondrial biogenesis, and systemic energy homeostasis (8). In T1D, sustained immune activation and insulin deficiency alter this myokine network through two routes: inflammatory cytokines act directly on the myocyte to modify myokine transcription, and the loss of contractile and oxidative stimuli that normally drive myokine release, including the exercise-induced IL-6 and irisin responses, reduces their secretion (9). The consequence is a sequence rather than a set of parallel changes: inflammatory signaling and impaired insulin action reduce mitochondrial fatty-acid oxidation, fatty-acid uptake continues unabated, and the resulting surplus of acyl-CoA is diverted toward ceramide and diacylglycerol synthesis and stored as intramyocellular lipid, with a progressive decline in oxidative capacity.

Systemic inflammation further intensifies these pathological processes. Elevated circulating cytokines such as TNF-α, interleukin-1β (IL-1β), and IL-6 impair insulin-receptor signaling and activate stress-responsive kinases and lipogenic transcriptional programs, reinforcing immune–metabolic dysregulation within skeletal muscle (10). Concomitantly, infiltration of macrophages and T cells into muscle tissue is thought to sustain local cytokine production, which in turn maintains the suppression of fatty-acid oxidation described above (11).

This review explores the immunometabolic mechanisms linking myosteatosis and T1D, with a focus on how inflammatory signaling, immune-cell muscle interactions, and dysregulated lipid metabolism may converge to impair glucose homeostasis. Particular emphasis is placed on nutrient-sensing pathways including AMP-activated protein kinase (AMPK), mechanistic target of rapamycin (mTOR), and peroxisome proliferator-activated receptors (PPARs), which integrate metabolic and immune cues within skeletal muscle. We further discuss emerging biomarkers and therapeutic strategies that target muscle lipid metabolism, inflammation, and mitochondrial health, with the goal of restoring immune-metabolic balance and preventing myosteatosis-related complications in T1D. A note on the nature of the evidence is warranted at the outset. Myosteatosis has not yet been systematically characterized in T1D, and much of the mechanistic literature on which we draw derives from T2D, obesity and ageing. The framework advanced here is therefore integrative and hypothesis-generating rather than confirmatory. We regard this imbalance as informative in its own right: throughout the review we identify where T1D-specific evidence is absent, and we consolidate these gaps in Section 6.1 to define the studies required to test the model. The mechanistic links proposed below should accordingly be read as plausible and internally consistent extrapolations awaiting direct validation in individuals with T1D.

## 2 From bioenergetic failure to lipid accumulation in T1D skeletal muscle

### 2.1 The initiating context in T1D: insulin deficiency, glycemic variability, and autoimmune activation

The mechanisms described in this section proceed from three conditions specific to T1D and present from the earliest stages of disease. The first is absolute insulin deficiency. Skeletal muscle accounts for the majority of insulin-stimulated glucose disposal (9), and in the absence of endogenous secretion its capacity to take up and oxidize glucose depends entirely on exogenous replacement, which is delivered peripherally rather than portally and in a pattern that does not reproduce physiological secretion (12). The second is glycemic variability. Even where mean HbA1c is well controlled, individuals with T1D experience repeated excursions between hyperglycemia and hypoglycemia, and it is the variability rather than the mean that most closely tracks markers of oxidative stress (5, 13). The third is sustained autoimmune activation, which in T1D persists long after β-cell destruction is complete and maintains a low-grade systemic inflammatory tone largely independent of adiposity (14).

These three inputs are not independent of one another, and they converge on a common target. Insulin deficiency limits flux through glycolysis and forces greater reliance on fatty-acid oxidation (6). Glycemic excursions impose repeated cycles of substrate overload followed by relative deprivation (5). Inflammatory cytokines act directly on muscle to suppress oxidative gene expression (15). Each places load on the mitochondrion, and it is at the mitochondrion that they become a single lesion.

For this reason we treat mitochondrial dysfunction not as the primary defect in T1D but as the proximal defect: the first point at which these disease-specific inputs converge into a unified bioenergetic failure, and the point from which the sequence described in the remainder of this review proceeds.

### 2.2 Mitochondrial dysfunction and oxidative stress

This convergence has structural and functional consequences that can be measured directly. Mitochondrial dysfunction is a defining feature of skeletal muscle in T1D and plays a pivotal role in the development of myosteatosis (16–21). Diminished PGC-1α activity, impaired oxidative phosphorylation, and excessive reactive oxygen species (ROS) generation disrupt mitochondrial homeostasis and drive lipid accumulation within myofibers (16, 20). The resulting decline in fatty-acid β-oxidation promotes the buildup of intramyocellular lipids and lipotoxic intermediates such as ceramides and diacylglycerols (DAGs), which further aggravate insulin resistance and inflammatory signaling (22).

Transmission electron microscopy and high-resolution respirometry of vastus lateralis biopsies from young adults with T1D have demonstrated altered mitochondrial ultrastructure and bioenergetics, including disrupted cristae morphology and reduced respiratory capacity (17, 21). These alterations reflect mitochondrial fragility and coincide with heightened oxidative stress, which activates NF-κB, JNK, and p38 MAPK pathways, amplifying inflammatory cytokine release and myokine dysregulation (23). These kinases converge on lipid handling. TNF-α-driven NF-κB activation impairs regulation of the muscle oxidative phenotype, reducing PGC-1α and its downstream oxidative targets in cultured muscle cells (24), and the relationship is reciprocal, since PGC-1α and PGC-1β in turn repress NF-κB signaling by reducing p65 phosphorylation (25); loss of PGC-1α therefore both follows from and further permits inflammatory signaling. In parallel, JNK phosphorylates insulin receptor substrate-1 at inhibitory serine residues including Ser307 (26), limiting insulin-stimulated glucose uptake and increasing reliance on the fatty-acid oxidation the mitochondrion cannot deliver. Suppression of AMPK activity and down-regulation of SIRT1/3 have been proposed to impair redox balance and blunt adaptive mitochondrial biogenesis. This has been characterized in T2D and animal models rather than in T1D muscle, where neither AMPK activity nor sirtuin expression has been directly quantified (23, 27, 28).

Beyond bioenergetic failure, mitochondrial injury provokes innate immune activation. Damaged organelles release damage-associated molecular patterns (DAMPs) including mitochondrial DNA (mtDNA), cardiolipin, and oxidized mitochondrial peptides that engage Toll-like receptors 2/4 (TLR2/4) and the NLRP3 inflammasome, initiating cytokine responses that act back on the same mitochondria, as described below (29, 30). This cascade culminates in caspase-1-dependent IL-1β release. IL-1β acts back on the myocyte through NF-κB p65, which is required for cytokine-induced myotube atrophy (31), so that inflammasome activation initiated by mitochondrial damage feeds into the same signaling that suppresses the oxidative phenotype. This closes the first of two feedback loops described in this review: mitochondrial damage generates the DAMPs that drive cytokine release, and those cytokines act back on the mitochondrion to further increase ROS production and inhibit lipid oxidation (32).

**Figure 1:**
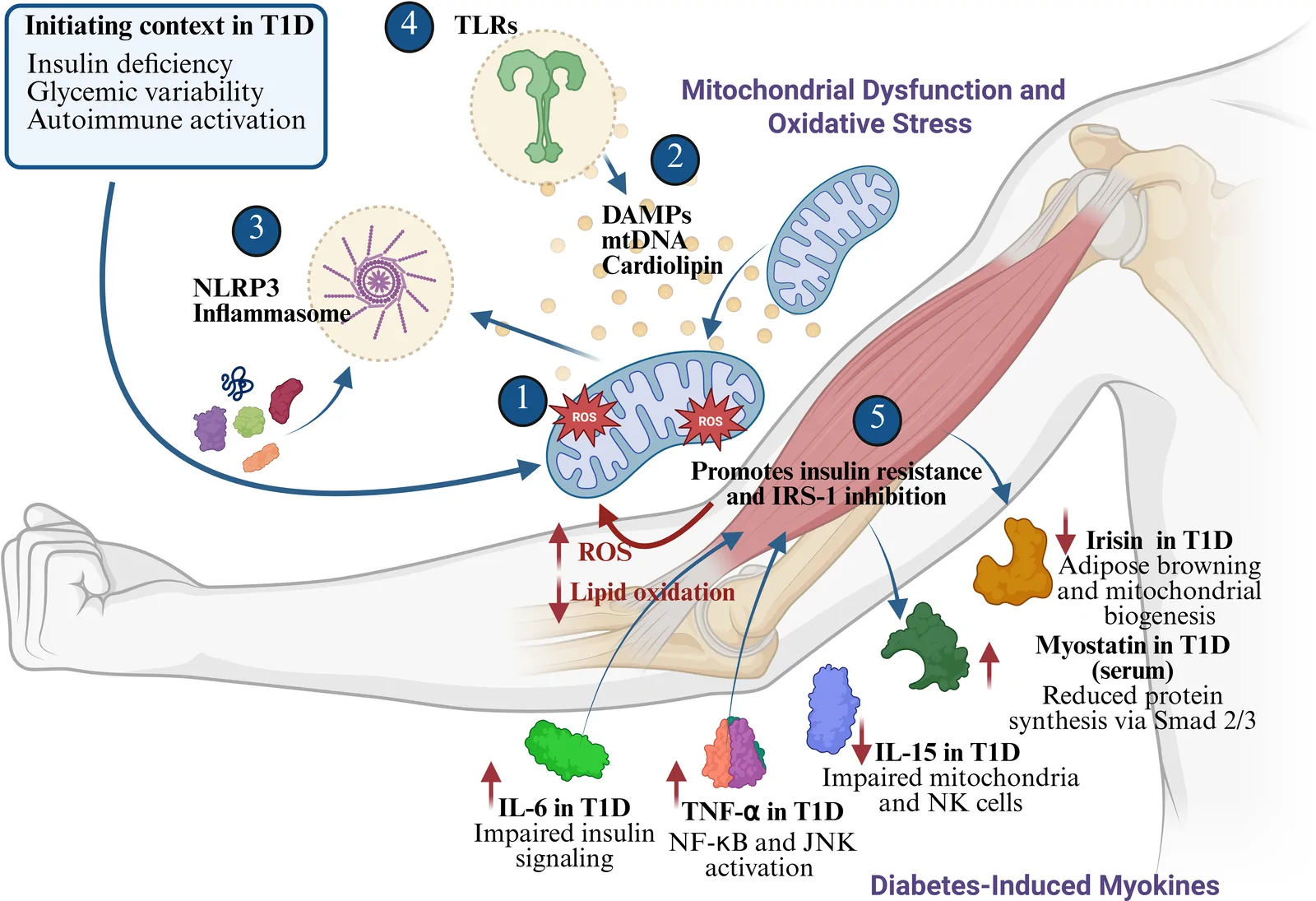
Mitochondrial dysfunction and oxidative stress in T1D skeletal muscle. Insulin deficiency, glycemic variability, and autoimmune activation converge on the mitochondrion (step 1), where transmission electron microscopy and high-resolution respirometry of vastus lateralis biopsies from young adults with T1D have demonstrated disrupted cristae morphology and reduced respiratory capacity, accompanied by diminished PGC-1α activity, impaired oxidative phosphorylation, and excessive reactive oxygen species (ROS) generation. Damaged mitochondria release damage-associated molecular patterns (DAMPs) including mitochondrial DNA (mtDNA) and cardiolipin (step 2), which engage the NLRP3 inflammasome (step 3) and Toll-like receptors (step 4), culminating in cytokine release that promotes insulin resistance and inhibition of insulin receptor substrate-1 (step 5). The red arrow indicates the resulting self-reinforcing loop, in which immune activation returns to the mitochondrion to further increase ROS production and reduce lipid oxidation. Dashed outlines denote steps demonstrated *in vitro* or extrapolated from other metabolic disease; the mitochondrial ultrastructural and respiratory findings have been demonstrated directly in T1D skeletal muscle. The lower panel summarizes myokines dysregulated in T1D. DAMP, damage-associated molecular pattern; IRS-1, insulin receptor substrate-1; mtDNA, mitochondrial DNA; NK, natural killer; NLRP3, NOD-like receptor family pyrin domain-containing 3; ROS, reactive oxygen species; T1D, type 1 diabetes; TLR, Toll-like receptor; TNF-α, tumor necrosis factor-alpha.

In individuals with T1D, glycemic variability contributes to these abnormalities through a mechanism distinct from that of sustained hyperglycemia. Fluctuating glucose concentrations activate protein kinase C, which drives assembly and activation of NADPH oxidase, and the resulting superoxide generation exceeds that produced by constant high glucose at the same mean concentration (33). Because each excursion imposes a fresh oxidative burden rather than allowing adaptation, variability rather than mean glycemia is the parameter most closely associated with markers of oxidative stress (5, 13). The consequent inability to switch efficiently between glucose and fatty-acid oxidation perpetuates metabolic rigidity and sustained oxidative stress (17, 19). Mitochondria therefore occupy the position at which immune activation, oxidative damage, and lipid accumulation converge in T1D skeletal muscle (34, 35) (**Figure 1**). It should be noted that the direct evidence for these specific mitochondrial defects derives predominantly from small cross-sectional studies; longitudinal data establishing causal directionality between mitochondrial dysfunction and intramyocellular lipid accumulation in T1D remain limited.

### 2.3 Impaired fatty-acid oxidation and the accumulation of lipotoxic intermediates

Mitochondrial dysfunction alters muscle lipid handling before it alters muscle mass, and the intermediate step is a mismatch between fatty-acid uptake and fatty-acid oxidation. Under normal conditions, fatty acids entering the myocyte are esterified to acyl-CoA by long-chain acyl-CoA synthetases (ACSL), transported into the mitochondrion by carnitine palmitoyltransferase 1(CPT1), and oxidized (36). When fatty-acid delivery exceeds the capacity of the tricarboxylic acid (TCA) cycle to consume the acetyl-CoA generated, β-oxidation proceeds but does not run to completion (36). Incomplete β-oxidation generates chain-shortened acylcarnitines that are exported from the mitochondrion rather than fully oxidized, and their appearance in plasma provides a direct readout of this mismatch (37, 38).

The lipid that accumulates is only partly inert. Triacylglycerol stored in cytosolic droplets is metabolically neutral and is found at high concentrations in the trained muscle of endurance athletes, the so-called athlete’s paradox (39, 40). What distinguishes pathological from physiological intramyocellular lipid is the accompanying pool of bioactive intermediates (41, 42). When acyl-CoA supply exceeds the combined capacity of oxidation and triacylglycerol synthesis, flux is diverted toward ceramide and diacylglycerol synthesis (43). Ceramides, particularly the C16:0 and C18:0 species, suppress Akt activation through protein phosphatase 2A (PP2A) and atypical protein kinase Cζ (aPKCζ), reducing insulin-stimulated glucose uptake (22, 44). Diacylglycerols activate protein kinase Cθ (PKCθ), which phosphorylates insulin receptor substrate-1 at inhibitory serine residues (45). Both reduce glucose disposal, which in turn increases reliance on the fatty-acid oxidation that the mitochondrion cannot deliver, closing a second loop that operates on substrate selection rather than on immune signaling.

This loop is the point at which a bioenergetic problem becomes an immunological one. Ceramides and their metabolites engage Toll-like receptor 4 and promote NLRP3 inflammasome assembly (5, 46), while mitochondrial DNA released from damaged organelles acts as a damage-associated molecular pattern (29). Lipid accumulation thereby ceases to be a passive consequence of impaired oxidation and becomes an active inflammatory stimulus.

The lipotoxic sequence described here is established in obesity, T2D, and animal models. Direct biopsy evidence in T1D muscle remains limited, and the pathway is presented as inferred rather than demonstrated in this population.

### 2.4 Failure of nutrient sensing: AMPK, mTORC1, and PPAR

The AMPK-mTOR-PPAR signaling axis serves as a central regulatory hub that integrates energy metabolism, mitochondrial function, and immune responses within skeletal muscle. Dysregulation of this axis is proposed here as a central feature of myosteatosis in T1D. It is supported by mechanistic data from hyperglycemic and lipotoxic models but has not been directly quantified in T1D muscle biopsy material.

AMPK, the key cellular energy sensor, is frequently suppressed under conditions of chronic hyperglycemia and oxidative stress (47–49). Reduced AMPK activity blunts fatty-acid oxidation and autophagic flux, leading to the accumulation of lipid droplets and lipotoxic intermediates within myofibers. This metabolic stagnation not only exacerbates lipid deposition but also permits sustained activation of pro-inflammatory signaling cascades, reinforcing the cycle of myosteatotic remodeling.

**Figure 2:**
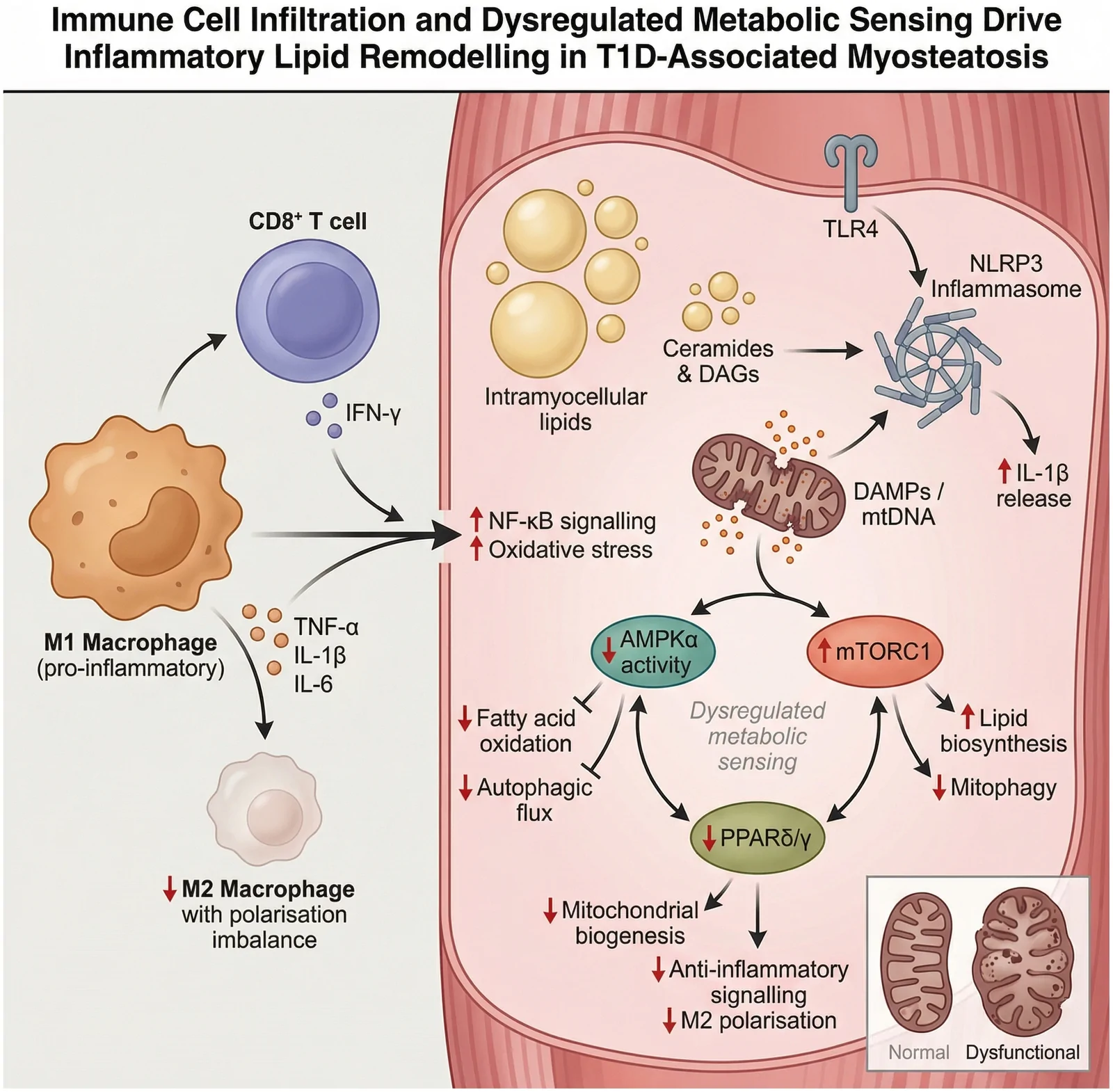
Proposed model: immune cell infiltration and dysregulated metabolic sensing driving inflammatory lipid remodeling in T1D-associated myosteatosis. Two-zone schematic illustrating how extracellular immune infiltration and intracellular metabolic sensor dysregulation are proposed to converge to sustain myosteatosis in T1D skeletal muscle. In the left extracellular zone (gray background), pro-inflammatory M1-polarised macrophages release TNF-α, IL-1β, and IL-6, while CD8^+^ T cells secrete IFN-γ; both cell types converge on the sarcolemma to amplify NF-κB signaling and oxidative stress within the fiber interior. A concurrent M2 macrophage polarization imbalance (shown as a faded, attenuated cell) impairs lipid clearance and anti-inflammatory resolution. In the right intracellular zone (pink background), accumulating intramyocellular lipids (gold droplets) generate ceramides and DAGs, which engage TLR4 at the sarcolemmal surface (upper right), activating the NLRP3 inflammasome and driving IL-1β release. A stressed mitochondrion releases DAMPs including mtDNA (orange particles), which feed back into TLR4 and NLRP3 activation, consolidating a self-amplifying inflammatory loop. These converging signals disrupt three interdependent metabolic sensors shown as a central regulatory cycle: AMPKα activity is suppressed (teal oval, ↓), impairing fatty-acid oxidation and autophagic flux; mTORC1 is elevated (coral oval, ↑), promoting lipid biosynthesis and inhibiting mitophagy; and PPARδ/γ is down-regulated (olive oval, ↓), reducing mitochondrial biogenesis, anti-inflammatory signaling, and M2 macrophage polarization capacity. This dysregulated metabolic sensing cycle sustains the immunometabolic niche that promotes progressive lipid deposition. The lower right inset illustrates the ultrastructural consequence of these combined insults, contrasting normal mitochondrial cristae architecture with the disorganized, swollen appearance of dysfunctional mitochondria in T1D skeletal muscle. Red ↑ and ↓ symbols denote relative increases and decreases, respectively, in T1D. Inhibitory connections are denoted by flat-headed bars. AMPKα, AMP-activated protein kinase alpha subunit; DAG, diacylglycerol; DAMP, damage-associated molecular pattern; IFN-γ, interferon-gamma; mtDNA, mitochondrial DNA; mTORC1, mechanistic target of rapamycin complex 1; NF-κB, nuclear factor kappa B; NLRP3, NOD-like receptor family pyrin domain-containing 3; PPARδ/γ, peroxisome proliferator-activated receptor delta/gamma; T1D, type 1 diabetes; TLR4, Toll-like receptor 4; TNF-α, tumor necrosis factor-alpha.

Conversely, mTOR complex 1 (mTORC1) is thought to be chronically activated by exogenous insulin exposure and glucose fluctuations, a mechanism proposed to drive anabolic resistance and increased cytokine production, although this has not been directly demonstrated in T1D muscle (50). Persistent mTORC1 signaling disrupts mitophagy and enhances lipid biosynthesis, thereby aggravating ectopic lipid accumulation and impairing mitochondrial quality control. Complementing these disruptions, PPARδ and PPARγ nuclear receptors that govern mitochondrial biogenesis and anti-inflammatory lipid metabolism are consistently down-regulated in T1D muscle (51–53). Their suppression limits PGC-1α expression, diminishes lipid utilization, and removes trans-repressive control over inflammatory gene networks, further intensifying immune activation and oxidative stress (54, 55). Restoring this metabolic-sensing triangle represents a promising therapeutic target for T1D-associated myosteatosis. Interventions such as pharmacological AMPK activators (e.g., metformin analogues), PPARδ/γ modulators, structured exercise, and lipid-modifying strategies may collectively enhance fat oxidation, mitochondrial turnover, and immune-metabolic balance, thereby reversing muscle lipid infiltration and improving systemic metabolic health (**Figure 2**).

## 3 Immune amplification and the transition to myosteatosis

As discussed earlier, mitochondrial dysfunction and oxidative stress create a permissive environment for lipid accumulation, inflammatory cytokine signaling, and altered myokine secretion. These changes reshape skeletal muscle from a metabolic sink into an immunometabolic organ that actively contributes to systemic inflammation and metabolic inflexibility. The resulting state - myosteatosis - reflects the convergence of immune activation, lipid remodeling, and impaired mitochondrial resilience.

### 3.1 Inflammatory signatures in T1D-associated myosteatosis

The lipotoxic intermediates generated in Section 2.3 are not metabolically inert; they act as ligands for innate immune receptors, and the inflammatory consequences are considered here. Unlike T2D, T1D is defined by persistent autoimmune activation that endures long after β-cell destruction. This sustained immune stimulation maintains chronically elevated levels of IL-6, TNF-α, and IL-1β, even in the absence of obesity, establishing a systemic inflammatory milieu that disrupts insulin signaling, suppresses oxidative metabolism, and promotes lipid deposition within myofibers, the defining hallmark of myosteatosis (56).

Among these cytokines, IL-6 and TNF-α exert dual metabolic and immunologic effects. Their chronic elevation impairs glucose uptake through IRS-1 inhibition while simultaneously enhancing peripheral lipolysis, thereby increasing free-fatty-acid flux to skeletal muscle (43, 57). The ensuing lipid overload supplies the ceramide and DAG pool described in Section 2.3, which engages TLR4 and the NLRP3 inflammasome within the myofiber (15, 46, 58, 59). TLR4 engagement sustains NF-κB activity, which maintains suppression of PGC-1α and the fatty-acid oxidation program, so that the lipid surplus generating these intermediates is not cleared.

Clinical and translational studies corroborate these mechanistic links: individuals with T1D and poor glycemic control exhibit elevated circulating myostatin, CRP, IL-6, and TNF-α (13, 14, 60, 61). Whether these systemic markers track muscle oxidative capacity or intramuscular fat content in T1D has not been established; the relationship is inferred from studies in obesity and T2D. In youth with early-onset T1D, this persistent inflammatory state, coupled with dysregulated lipid metabolism, may lay the foundation for premature myosteatosis and progressive decline in muscle metabolic health, although prospective longitudinal data directly linking early glycemic dysregulation to intramyocellular lipid accumulation in T1D youth are currently scarce (6, 7, 62).

### 3.2 Myokines and immune cross-talk in T1D

Beyond the cytokines discussed above, skeletal muscle itself contributes to this inflammatory milieu through its secretome. Skeletal muscle-derived myokines orchestrate systemic communication between muscle, adipose tissue, and the immune system, thereby regulating energy metabolism and inflammatory tone (63). In T1D, chronic autoimmune activation and recurrent glycemic excursions are proposed to alter myokine secretion, contributing to the lipid infiltration, mitochondrial dysfunction, and low-grade inflammation characteristic of myosteatosis.

Among the pro-inflammatory mediators, IL-6 and TNF-α play central roles (63). IL-6, transiently elevated during physical activity, exerts anti-inflammatory and insulin-sensitizing effects in healthy muscle (64). Yet chronic IL-6 exposure impairs insulin signaling in skeletal muscle through JNK-mediated serine phosphorylation of insulin receptor substrate-1 (IRS-1), induction of suppressor of cytokine signaling 3 (SOCS3), and increased PTP1B activity (65), which reduces insulin-stimulated glucose uptake and shifts substrate reliance toward fatty acids at a time when their oxidation is already impaired. Whether this operates in T1D is uncertain: SOCS3 mRNA is elevated in the skeletal muscle of individuals with T2D and correlates with reduced insulin-stimulated glucose uptake, but was found to be normal in individuals with T1D (66), suggesting the pathway may be driven by factors other than hyperglycemia alone. This shift promotes intramyocellular lipid accumulation and mitochondrial inefficiency.

Similarly, TNF-α, up-regulated by both autoimmune and metabolic stress, drives myosteatotic remodeling through several converging mechanisms: enhancing peripheral lipolysis, increasing free-fatty-acid flux to muscle, and activating NF-κB and JNK pathways that suppress insulin signaling and β-oxidation (67). TNF-α acts through TNFR1 to activate IKKβ and JNK, which phosphorylate IRS-1 at inhibitory serine residues, and through NF-κB to suppress PGC-1α transcription; the muscle therefore loses both insulin-stimulated glucose disposal and the transcriptional capacity for fatty-acid oxidation at the same time. Elevated TNF-α fosters ceramide accumulation and oxidative stress, feeding both loops (10, 68).

Counterbalancing these catabolic effects, certain myokines such as IL-15, myostatin, and irisin modulate the anabolic and oxidative capacity of muscle. IL-15 signals through the IL-15Rα/IL-2Rβ/γc complex on the myocyte, activating JAK1/JAK3 and STAT5 to increase PGC-1α and PPARδ expression, and thereby mitochondrial biogenesis and fatty-acid oxidation (69). The same receptor complex, presented in trans, sustains natural-killer and CD8^+^ T-cell survival, which is why IL-15 couples the oxidative and immune arms of muscle function. Experimental restoration of IL-15 signaling improves mitochondrial function and insulin sensitivity in cell and rodent models (70–72). IL-15 also modulates fibro-adipogenic progenitors during muscle regeneration in mice (73), which is the only route by which it has been linked directly to the cell population that gives rise to intramuscular adipocytes. Whether IL-15 is suppressed in T1D skeletal muscle, and whether this contributes to anabolic resistance, has not been established, and the link is proposed here on the basis of these model.

Conversely, myostatin binds the activin type IIB receptor and signals through ALK4/ALK5 to phosphorylate Smad2 and Smad3, which inhibits Akt/mTOR/p70S6K signaling and reduces protein synthesis; this has been demonstrated in human primary myotubes *in vitro* (74). Its effect on adipogenic programming is directionally inconsistent: in human bone marrow-derived mesenchymal stem cells and preadipocytes, myostatin reduced lipid accumulation and down-regulated PPARγ and C/EBPα through Smad3-Wnt/β-catenin cross-talk (75), while other *in vitro* systems report the opposite. The myostatin contribution to intramuscular lipid accumulation is therefore presented here as unresolved rather than established. Myostatin inhibition improves muscle regeneration and mitochondrial efficiency in preclinical models, including an insulin-deficient mouse model of T1D (76, 77). In adults with T1D, serum myostatin is elevated but intramuscular myostatin protein is not, a dissociation whose implications for measurement are taken up with the other candidate markers.

Finally, irisin, cleaved from Fibronectin type III domain-containing protein 5 (FNDC5), links muscular activity to systemic metabolism by promoting adipose tissue browning, mitochondrial biogenesis, and anti-inflammatory signaling. Irisin release depends on PGC-1α-driven FNDC5 transcription during contraction, so reduced physical activity lowers irisin at source rather than through a signaling defect. This makes irisin a marker of the contractile stimulus itself, which is relevant in T1D where exercise participation is frequently limited by hypoglycemia risk (78–80). In T1D, diminished irisin levels particularly among sedentary individuals further compromise oxidative metabolism and immune regulation, fostering conditions conducive to intramuscular lipid deposition (81).

Collectively, these myokines constitute an interconnected immune–metabolic network that governs muscle lipid handling, mitochondrial function, and systemic glucose regulation (82, 83). In T1D, dysregulation of this network shifts the balance from metabolic adaptation to chronic inflammation, driving the lipid-laden, mitochondrially stressed phenotype that defines myosteatosis.

### 3.3 Immune cell infiltration of skeletal muscle

Soluble mediators alone do not account for the muscle phenotype; cellular infiltration of the tissue is the next step in the sequence. Even in lean individuals, T1D is thought to involve low-grade skeletal muscle inflammation, with infiltration of macrophages and CD8^+^ T cells into the perimysium and endomysium reported in metabolic disease models and inferred for T1D (7, 84). These immune cells release pro-inflammatory cytokines that activate NF-κB signaling, enhance oxidative stress, and impair mitochondrial lipid oxidation, thereby promoting intramyocellular lipid accumulation (85).

Within this environment, macrophage polarization is skewed toward the M1 (pro-inflammatory) phenotype, driving secretion of TNF-α, IL-1β, and IL-6, which suppress fatty-acid oxidation and stimulate ceramide synthesis (84). In contrast, M2-like macrophages (anti-inflammatory), normally responsible for lipid clearance, oxidative repair, and tissue remodeling are diminished in both number and activity, limiting resolution of inflammation and metabolic recovery (86). CD8^+^ T cells further amplify this inflammatory loop through IFN-γ release, which reinforces M1 polarization and down-regulates PGC-1α-dependent mitochondrial biogenesis, compounding oxidative deficits (55, 87, 88).

Together, this immune-metabolic interplay is proposed to create a lipid-inflammatory niche within skeletal muscle that sustains myosteatosis and contributes to the loss of metabolic flexibility in long-standing T1D (7, 21). Studies combining biopsy immunophenotyping with metabolic profiling in well-characterized T1D cohorts are required to establish whether this component of the model operates in this disease.

### 3.4 Neurogenic contributions: peripheral neuropathy and muscle denervation

Several studies have demonstrated a further route to intramuscular fat accumulation, one that acts on muscle from outside rather than within, through loss of motor innervation (89, 90). Denervation is among the most reproducible causes of fatty infiltration in the wider myosteatosis literature, documented in ageing, spinal cord injury, motor neuron disease and traumatic nerve injury (91, 92). Its relevance to T1D is direct: distal symmetrical polyneuropathy affects at least one in five individuals with T1D after twenty years of disease (93), rising towards a third at longer durations in the DCCT/EDIC cohort (94). Any account of muscle quality in long-duration T1D that omits denervation is therefore incomplete.

Motor involvement in diabetic polyneuropathy is clinically underappreciated because sensory symptoms dominate the presentation, but it is readily demonstrable with quantitative techniques. Length-dependent loss of motor axons and reduced motor unit number are documented in human diabetic polyneuropathy, affecting distal muscles first (95, 96). Isokinetic dynamometry reveals ankle and knee weakness in individuals with T1D, and this weakness is confined to those with polyneuropathy: individuals with long-duration T1D and intact nerve function retain normal strength (97, 98). Stereological MRI has shown corresponding atrophy of the lower leg and foot musculature that parallels neuropathy severity (99, 100) and accelerates on longitudinal follow-up (101), and quantitative MRI in participants with diabetic polyneuropathy shows fat accumulation that is most pronounced in the plantar flexors (102). In cohorts with T2D and peripheral neuropathy, intermuscular and intramuscular adipose tissue is markedly increased and tracks with reduced plantar flexion torque, shorter walking distance and poorer physical function (103, 104). Fat accumulation in this setting therefore carries functional consequences that are additive to those attributable to lipotoxicity.

The cellular route from denervation to fat is now reasonably well defined, and it converges on the signaling already described above. Intramuscular adipocytes derive principally from muscle-resident fibro-adipogenic progenitors, a PDGFRα-positive mesenchymal population that remains quiescent in healthy muscle and adopts an adipogenic fate under pathological conditions (89, 90). Denervation drives progressive accumulation of these progenitors and, importantly, does so without the macrophage infiltration and satellite-cell-mediated regeneration that follow acute injury. Denervation-activated progenitors instead display sustained STAT3 activation and secrete elevated IL-6 and inactivating this axis limits atrophy and fibrosis in models of denervation and motor neuron disease (91). This represents convergence rather than competition: IL-6 and STAT3 are already central to the cytokine network described in Section 3.2, so denervation and immunometabolic stress plausibly act on a shared downstream effector population. Chronic hyperglycemia and lipotoxic stress may prime fibro-adipogenic progenitors towards an adipogenic fate while progressive denervation supplies the activating signal, generating more fat infiltration than either insult would produce alone.

Two practical implications follow. First, the anatomical distribution of infiltration may help separate the contributions. Neurogenic fat accumulation follows the length-dependent, distally predominant pattern of diabetic polyneuropathy and is most marked in the intrinsic foot muscles and plantar flexors (100, 102), whereas much of the metabolic literature has quantified fat in proximal thigh compartments. Studies of muscle fat in T1D should therefore report compartment-specific measures and stratify by neuropathy status rather than treating whole-limb fat fraction as a single phenotype. Second, neuropathy status is a plausible effect modifier for exercise-based interventions, since denervated muscle has reduced capacity to respond to resistance training and concurrent sensory loss alters the safety profile of weight-bearing exercise.

## 4 Emerging biomarkers of myosteatosis in T1D

Each step of the cascade described in Sections 2 and 3 generates measurable products, and the biomarkers considered here are grouped accordingly. Identifying reliable biomarkers that capture early lipid infiltration and metabolic remodeling within skeletal muscle in individuals with T1D is crucial for improving both clinical risk stratification and mechanistic understanding of myosteatosis. Such biomarkers encompass inflammatory cytokines, muscle-derived myokines, metabolic regulators, and imaging- or omics-based indicators of muscle lipid content, mitochondrial function, and immune activation.

### 4.1 Inflammatory and myokine-based biomarkers

The inflammatory and myokine mechanisms established in Sections 3.1 and 3.2 yield the most accessible candidate markers. Circulating inflammatory cytokines such as IL-6, TNF-α, and IL-1β are elevated in individuals with T1D, particularly under conditions of poor glycemic control (13, 14). Beyond their catabolic effects, these cytokines disrupt lipid oxidation and stimulate intramyocellular lipid accumulation by the mechanisms described in Section 3.1, making them candidate markers of the inflammatory contribution to myosteatosis (13, 65, 67). Elevated C-reactive protein (CRP) and serum amyloid A (SAA) further indicate chronic low-grade inflammation in T1D (14); their relationship to muscle lipid infiltration is inferred from obesity and T2D and has not been examined in this population. Within this inflammatory milieu, muscle-derived myokines exert complementary yet divergent influences on lipid remodeling, and their value as markers depends on whether circulating concentrations report on the muscle compartment. Myostatin illustrates the difficulty. In adults with T1D, serum myostatin is elevated relative to controls and more markedly so in women than in men, yet intramuscular myostatin protein does not differ and circulating concentrations correlate with neither HbA1c nor disease duration (61). Circulating myostatin therefore does not appear to report intramuscular myostatin expression in this population, and its lack of association with glycemic control or disease duration limits its use for stratification. The mechanistic basis for its inclusion is set out in Section 3.2 and rests on preclinical models rather than on human muscle data. Follistatin, a natural antagonist of myostatin, has been proposed as a protective counter-regulator supporting mitochondrial function and limiting lipid accumulation within myofibers (105), although it has not been measured in relation to muscle lipid content in T1D.

IL-15 has been proposed as a marker of the same axis, but the evidence in T1D is weaker still. Its metabolic and immunoregulatory functions are set out in Section 3.2; the question here is whether circulating IL-15 reflects them. Muscle IL-15 acts substantially through trans-presentation of a membrane-bound IL-15Rα complex rather than as a freely diffusing cytokine, so plasma concentrations are an indirect surrogate for local signaling. Neither circulating nor intramuscular IL-15 has been measured against muscle lipid content in T1D. IL-15 is therefore included as a mechanistically motivated candidate rather than as a marker with demonstrated performance, and the direction of change shown for it in **Table 1** is inferred from the models described in Section 3.2 rather than measured in this population.

**Table 1:** Summary of emerging biomarkers of myosteatosis in T1D.

Category	Representative biomarkers	Primary biological role	Direction of change in T1D	Clinical/pathophysiological relevance	References
Inflammatory cytokines	IL-6, TNF-α, IL-1β, CRP, serum amyloid A	Promote NF-κB/JNK signaling, impair insulin action, and sustain chronic inflammation	↑ (chronic elevation; measured in T1D)	Reflect systemic low-grade inflammation in T1D; associations with muscle lipid content and oxidative capacity are inferred from obesity and T2D	(13, 14, 65, 66)
Muscle-derived myokines	Myostatin, follistatin, IL-15, irisin	Regulate muscle growth, mitochondrial biogenesis, and immune tone	Myostatin ↑ and irisin ↓ (measured in T1D); follistatin and IL-15 ↓ (inferred)	Imbalance between anabolic and catabolic myokines is proposed to promote lipid accumulation and metabolic inflexibility; circulating myostatin does not track intramuscular myostatin in T1D	(61, 69, 74, 81, 105)
Lipid-derived mediators	Ceramides (C16:0, C18:0), DAGs, oxidized lipids (MDA, 4-HNE)	Induce TLR4/NLRP3 activation, inhibit Akt-mTOR, and generate oxidative stress	↑ (plasma ceramides measured in T1D)	Proposed indicators of intramyocellular lipotoxicity; prognostic for cardiovascular events in T1D, but the relationship to muscle lipid content has not been examined	(22, 43, 109, 111, 112)
Metabolic intermediates	Acylcarnitines (C14:1, C16, C18:1), lactate, β-hydroxybutyrate	Reflect mitochondrial β-oxidation efficiency and substrate switching	↑ (medium/long-chain species; lactate; ketones)	Signal incomplete fatty-acid oxidation and metabolic inflexibility; correlate with poor glycemic control	(36, 38, 106, 107)
Mitochondrial-stress markers	mtDNA (free and EV-bound), PGC-1α, SIRT1/3, NAD^+^	Mediate mitochondrial biogenesis, redox homeostasis, and mitophagy	mtDNA ↑, NAD^+^/SIRT ↓	Elevated mtDNA is associated with inflammasome activation; NAD^+^ decline indicates defective energy and redox balance	(113–116)
Omics-based signatures	Transcriptomic: ACSL1, CPT1A, PGC-1α, NLRP3; proteomic/metabolomic: lipid oxidation and cytokine modules	Integrate metabolic, inflammatory, and mitochondrial pathways	Not established in T1D; available muscle datasets derive from obesity and T2D	Would enable molecular phenotyping of myosteatosis and identification of therapeutic targets	(37, 121, 122)
Imaging-derived indicators	MRI fat-fraction mapping, ¹H/³¹P-MRS, ultrasound elastography	Quantify intramyocellular lipid content, oxidative capacity, and fibrosis	↑ fat fraction, ↓ phosphocreatine recovery (measured in T1D)	Provide non-invasive evaluation of muscle lipid remodeling and metabolic health; no consensus threshold defines myosteatosis on any modality	(102, 118–120)

↑, increased; ↓, decreased, relative to individuals without diabetes.

### 4.2 Metabolic and mitochondrial biomarkers

The bioenergetic and lipotoxic processes described in Sections 2.2 and 2.3 generate circulating and tissue-based markers that can be measured non-invasively. Acylcarnitine profiling has emerged as a sensitive indicator of mitochondrial β-oxidation efficiency. In insulin-resistant states, elevations in medium- and long-chain acylcarnitines (e.g., C14:1, C16, C18:1) consistently reflect incomplete fatty-acid oxidation and correlate with both reduced insulin sensitivity and increased intramyocellular lipid deposition (36, 38). In T2D, these intermediates track impaired lipid utilization and mitochondrial stress (38); whether they perform the same role in T1D-associated myosteatosis has not been directly tested.

Secondary metabolic markers reinforce this pattern of mitochondrial inefficiency. Accumulation of lactate, particularly during mild exertion, indicates a compensatory shift toward glycolytic reliance; lactate clearance during exercise is impaired in T1D and is further suppressed by hyperglycemia (106). Hyperketonemia, even in the absence of ketoacidosis, signals excessive peripheral lipolysis and unrestrained fatty-acid oxidation driven by insulinopenia (107). Although transient increases in ketone bodies may provide alternative fuel, sustained hyperketonemia in T1D is associated with increased lipid peroxidation and depletion of reduced glutathione, effects attributable principally to acetoacetate rather than to β-hydroxybutyrate (107, 108). Within this altered lipid landscape, ceramides and DAGs are the lipotoxic intermediates most readily measured in blood (109). Their mechanistic actions are set out in Section 2.3; the question here is whether circulating concentrations report on the muscle compartment. In adults with obesity and T2D, plasma ceramides are elevated and track the severity of insulin resistance (110), which is the basis for proposing them as markers of lipotoxic stress. In T1D, specific ceramide species and ratios predict cardiovascular events and all-cause mortality over six years in a cohort with long-standing disease (111). This establishes prognostic value in T1D but not specificity for muscle, since the relationship between circulating ceramides and skeletal muscle lipid content has not been examined in this population.

Markers of oxidative damage further capture the redox dimension of myosteatosis. Malondialdehyde (MDA) and 4-hydroxynonenal (4-HNE), products of lipid peroxidation, form adducts with mitochondrial proteins and DNA, precipitating respiratory-chain injury, apoptotic signaling, and progressive loss of muscle metabolic integrity (112). Complementing these findings, Circulating mtDNA, detected either freely or encapsulated within Extracellular Vesicles (EV), serves as a marker of mitochondrial injury and immune activation (113, 114). Elevated levels have been reported in diabetes and are proposed to correlate with NLRP3 inflammasome activation and impaired mitochondrial biogenesis, although this has not been characterized in T1D skeletal muscle. EV-associated mtDNA may further act as a paracrine danger signal.

Finally, nicotinamide adenine dinucleotide (NAD^+^) metabolism constitutes a pivotal node connecting mitochondrial function, redox balance, and immune regulation (115). NAD^+^ metabolism is dysregulated in both forms of diabetes, though by different routes: depletion is attributed principally to poly(ADP-ribose) polymerase activation in T1D and to AMPK inhibition in T2D, compounded in both by impaired nicotinamide salvage (116). Reduced NAD^+^ availability limits SIRT1 and SIRT3 activity, compromising mitophagy, lipid oxidation, and anti-inflammatory signaling (115). Circulating NAD^+^ and its precursors (e.g., nicotinamide riboside, nicotinamide mononucleotide) are therefore emerging as candidate biomarkers of mitochondrial decline (117), although they have not been evaluated in T1D-related myosteatosis.

**Figure 3:**
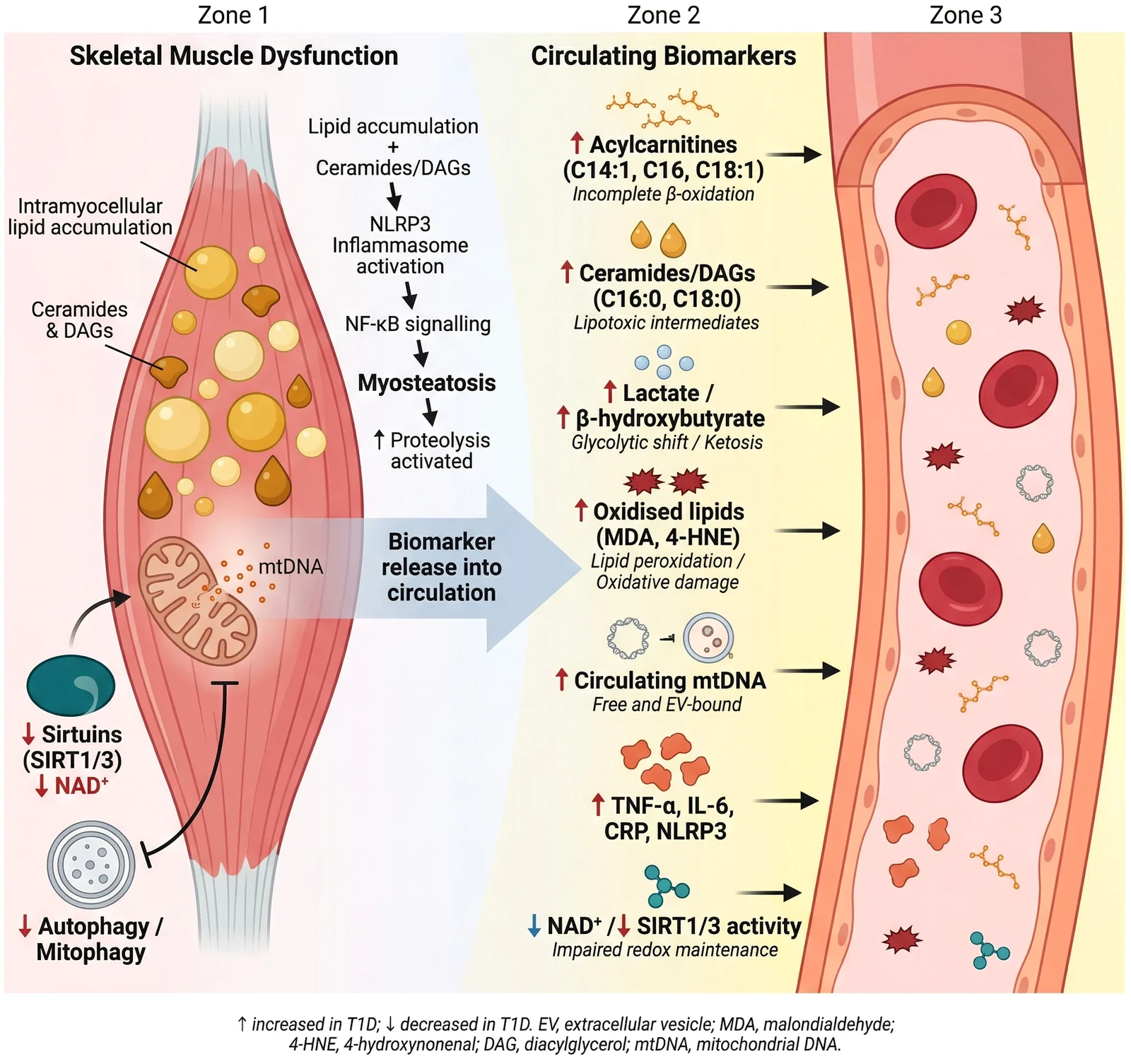
Circulating inflammatory, metabolic, and mitochondrial biomarkers reflecting skeletal muscle lipid remodeling in T1D-associated myosteatosis. Three-zone schematic linking intramuscular pathological events (Zone 1) to a characteristic blood-based biomarker signature (Zones 2 and 3) in T1D. In Zone 1 (skeletal muscle), lipid overload drives accumulation of intramyocellular lipids (gold droplets) and lipotoxic intermediates including ceramides and DAGs (amber deposits), which activate the NLRP3 inflammasome and NF-κB signaling, reinforcing myosteatosis and stimulating proteolysis. A stressed mitochondrion releases mtDNA (orange particles) that further amplify innate immune activation via a feed-forward loop. Concurrently, reduced sirtuin activity (SIRT1/3, teal node, ↓) and depleted NAD^+^ impair mitochondrial quality control, while suppressed autophagy and mitophagy (shown with inhibitory bars) prevent clearance of dysfunctional organelles, consolidating oxidative stress and metabolic inflexibility. A broad biomarker release arrow (Zone 1 to Zone 2) illustrates the translocation of muscle-derived signals into the systemic circulation. In Zone 2, seven circulating biomarker classes are depicted with their mechanistic subtitles: medium- and long-chain acylcarnitines (C14:1, C16, C18:1), reflecting incomplete fatty-acid β-oxidation; ceramides and DAGs (C16:0, C18:0), as lipotoxic intermediates; lactate and β-hydroxybutyrate, indicating a compensatory glycolytic shift and ketosis; oxidized lipids (MDA and 4-HNE), reflecting lipid peroxidation and oxidative damage; circulating mtDNA both free and encapsulated within extracellular vesicles, as markers of mitochondrial injury and paracrine inflammatory signaling; elevated TNF-α, IL-6, CRP, and NLRP3-associated mediators, capturing systemic inflammatory activation; and reduced NAD^+^ with diminished SIRT1/3 activity, reflecting impaired redox maintenance. Each biomarker class is represented by a corresponding icon and directed by a horizontal arrow into Zone 3, a cross-sectional blood vessel containing erythrocytes (deep rose-red biconcave structures) and freely circulating biomarker icons within the plasma compartment (pale yellow), confirming systemic detection. Red ↑ and ↓ symbols denote relative increases and decreases, respectively, in T1D. 4-HNE, 4-hydroxynonenal; CRP, C-reactive protein; DAG, diacylglycerol; EV, extracellular vesicle; MDA, malondialdehyde; mtDNA, mitochondrial DNA; NAD^+^, nicotinamide adenine dinucleotide; NF-κB, nuclear factor kappa B; NLRP3, NOD-like receptor family pyrin domain-containing 3; SIRT1/3, sirtuin 1/3; T1D, type 1 diabetes; TNF-α, tumor necrosis factor-alpha.

Collectively, these metabolic and mitochondrial biomarkers delineate the complex interplay between lipid handling, oxidative metabolism, and immune activation that underpins myosteatosis in T1D. Their incorporation into integrated imaging-omics frameworks may enable early detection of muscle lipid remodeling and guide the development of mechanistically targeted interventions to restore metabolic resilience (**Figure 3**).

None of the circulating markers discussed has been evaluated for diagnostic performance against a reference standard for myosteatosis in T1D. Reported associations are cross-sectional correlations rather than measures of discrimination, and no sensitivity, specificity, or area under the receiver operating characteristic curve has been established for any of them in this population. Several face practical constraints: ceramide and acylcarnitine quantification requires liquid chromatography-mass spectrometry rather than routine immunoassay, with limited inter-laboratory standardization, and circulating cell-free mtDNA is sensitive to pre-analytical handling. These markers should therefore be regarded as candidates for validation rather than as clinically deployable tests.

### 4.3 Imaging and omics approaches

Where circulating markers report on systemic processes, imaging addresses the tissue compartment in which the lipid actually accumulates. Traditional imaging modalities such as dual-energy X-ray absorptiometry (DXA) and magnetic resonance imaging (MRI) remain the gold standards for quantifying skeletal muscle mass. However, these techniques are limited in their ability to detect early compositional and metabolic alterations that define myosteatosis. Advances in magnetic resonance spectroscopy (^1^H and ^31^P-MRS), quantitative MRI, and ultrasound elastography now permit high-resolution assessment of intramyocellular lipid content, oxidative capacity, and fibrotic remodeling (118, 119). These approaches can distinguish lipid infiltration from true muscle loss, thereby providing more sensitive and functionally meaningful indicators of muscle metabolic health (120), although they have not yet been applied systematically in T1D cohorts. Beyond imaging, multi-omics profiling offers a route to characterizing the molecular networks underlying metabolic and inflammatory remodeling, though it has not yet been applied to T1D-associated myosteatosis. High-throughput transcriptomic, proteomic, and metabolomic analyses of muscle tissue and circulating compartments including plasma EVs and exosomes have identified distinct inflammatory, mitochondrial, and lipid-metabolic signatures.

Differential expression of genes involved in fatty-acid transport, including carnitine palmitoyl transferase 1A (CPT1A) and acyl-CoA synthetase long-chain family member 1 (ACSL1), mitochondrial biogenesis (PGC-1α, SIRT3), and immune activation (NLRP3, IL-6) reflects the intricate cross-talk between metabolic and immune pathways in diabetic skeletal muscle (121).

Consolidative lipidomic and metabolomic profiling further complements these findings by identifying ceramide species, acylcarnitine chains, and oxidized lipid intermediates as molecular fingerprints of lipotoxic stress and oxidative imbalance. When combined with advanced imaging tools such as MRI-based fat-fraction mapping and MRS-derived lipid quantification these omics-derived biomarkers enable precision phenotyping of early myosteatosis and provide dynamic measures for monitoring therapeutic response. The convergence of imaging and omics technologies thus offers a powerful, non-invasive framework for detecting muscle lipid remodeling and tracking immune-metabolic dysfunction in T1D (1, 122). Together, these approaches bridge molecular mechanisms with functional outcomes, paving the way toward personalized prevention and treatment strategies. A summary of key emerging biomarkers is presented in **Table 1**.

The mechanistic value of these approaches lies in their ability to separate steps of the cascade that circulating markers cannot distinguish. ¹H-MRS quantifies intramyocellular lipid directly and paired with ³¹P-MRS measurement of phosphocreatine recovery kinetics it can establish whether lipid accumulation precedes or follows the loss of oxidative capacity, which is the central unresolved question in the sequence proposed here. Dixon MRI separates intramuscular from intermuscular fat, a distinction that matters because the two compartments have different cellular origins: intramyocellular lipid reflects the substrate imbalance described in Section 2.3, whereas intermuscular adipose tissue derives from fibro-adipogenic progenitors and is the compartment expected to expand under denervation (Section 3.4). Compartment-specific imaging is therefore the only non-invasive means of distinguishing the metabolic from the neurogenic contribution. For omics, the relevant contribution is not signature discovery but discrimination between competing explanations: single-cell and spatial transcriptomics of muscle biopsy material would establish whether the inflammatory signal derives from resident myocytes or from infiltrating immune cells, a question that bulk expression data cannot resolve and that determines whether the immune-cell step of the model operates in T1D at all.

The imaging modalities discussed differ substantially in accessibility. Dixon-based MRI fat fraction is quantitative and reproducible but requires scanner time and analysis expertise; ¹H- and ³¹P-MRS remain largely confined to research centers; ultrasound elastography is portable and inexpensive but operator-dependent, with reproducibility yet to be established in muscle. No consensus threshold defines myosteatosis on any modality, which currently precludes case definition in clinical practice. Omics approaches remain exploratory in this context, with the available muscle datasets derived from obesity and T2D rather than T1D, and sample sizes insufficient for validation.

## 5 Therapeutic interventions targeting myosteatosis in T1D

### 5.1 Exercise and rehabilitation

Of the interventions considered, exercise acts furthest upstream, on the mitochondrial and nutrient-sensing steps described in Sections 2.2 and 2.4. Structured resistance and aerobic training remain the most effective non-pharmacological strategies for mitigating myosteatosis and restoring skeletal muscle metabolic flexibility in T1D. Exercise promotes mitochondrial biogenesis, enhances fatty-acid oxidation, and induces secretion of anti-inflammatory myokines such as IL-15, irisin, and transient exercise-induced IL-6. In trained individuals without diabetes, these adaptations limit intramuscular lipid deposition, improve insulin sensitivity, and reduce low-grade inflammation (40). Clinical studies in individuals with T1D show that high-intensity interval training (HIIT) and progressive resistance training increase lean mass and augment oxidative capacity (123–125). These effects are most pronounced when exercise is paired with personalized nutritional strategies and optimized insulin timing (126). Improvements in glucose variability and time-in-range further alleviate metabolic stressors that drive myosteatotic remodeling, underscoring the indirect glycemic benefits of structured training programs. Despite clear physiological advantages, glycemic lability and fear of hypoglycemia frequently limit exercise engagement in T1D. The use of continuous glucose monitoring, automated insulin delivery systems, and targeted pre-exercise carbohydrate intake is essential to balance safety with metabolic gains (7, 127, 128). Combined aerobic and resistance programs may confer additive benefit over either modality alone, although this has been examined in athletes rather than in individuals with T1D (129).

Neuropathy status also warrants consideration when prescribing exercise in this population. Denervation reduces motor unit number and the proportion of contractile tissue within affected muscles (95, 96), which may limit the hypertrophic and oxidative response to resistance training, and because up to half of diabetic peripheral neuropathies are asymptomatic, unrecognized loss of protective sensation places the insensate foot at risk during weight-bearing activity (93). Assessment of neuropathy status at baseline, with outcomes stratified accordingly, would strengthen future exercise trials in T1D.

### 5.2 Nutritional interventions

Nutritional strategies act principally on substrate supply, and therefore on the lipid-handling step described in Section 2.3, targeting restoration of metabolic flexibility, enhancement of lipid oxidation, and preservation of mitochondrial integrity. Adequate protein intake, with an emphasis on leucine-rich or leucine-enriched formulations, stimulates mTORC1 signaling and muscle protein synthesis and may help counteract diabetes-associated anabolic resistance. Acute studies in adolescents with T1D have shown that supplemental leucine improves whole-body protein metabolism, supporting the concept that targeted amino acid provision can partially normalize anabolic responses in this population (130). However, chronic inflammation, oxidative stress, and suboptimal insulin timing can blunt these benefits, underscoring the importance of aligning protein dosing with prandial insulin delivery to preserve glycemic stability while supporting muscle maintenance.

Omega-3 polyunsaturated fatty-acids (PUFAs) represent another promising adjunct owing to their anti-inflammatory and pro-resolving properties. Through activation of PPARα/δ and attenuation of NF-κB signaling, n-3 PUFAs promote lipid clearance, improve mitochondrial β-oxidation, and modulate vascular and neural complications. In T1D, randomized controlled trials have shown that high-dose omega-3 supplementation is safe and can improve early microvascular and neural outcomes, including peripheral nerve structure and function, even when effects on HbA1c are modest (131, 132). Emerging Mendelian randomization data further support a potential protective role of omega-3 status in T1D pathophysiology, although causal effects on muscle-specific endpoints such as myosteatosis have not yet been tested (133).

Micronutrient and ergogenic supplements may also contribute to preserving muscle quality in T1D and related metabolic states. Vitamin D deficiency is common in diabetes, and several trials and meta-analyses indicate that repletion can improve insulin sensitivity, β-cell function, or inflammatory profiles in subsets of deficient individuals, although results are heterogeneous and often confounded by baseline status and dose (134). Creatine supplementation, particularly when combined with resistance or aerobic training, has been shown to enhance GLUT4 translocation, improve glucose uptake, and augment gains in muscle performance in diabetes and insulin-resistant cohorts (135). Although dedicated trials in T1D are scarce, these mechanistic data support the rationale for carefully monitored creatine use as part of multimodal rehabilitation strategies, especially in individuals with low muscle mass or high functional demands.

Finally, emerging work on mitochondrial-targeted nutritional interventions offers an additional avenue to address early myosteatotic change. Augmentation of intracellular NAD^+^ pools via nicotinamide riboside (NR) or nicotinamide mononucleotide (NMN) has been shown in preclinical models to enhance SIRT1/3-dependent mitochondrial quality control, reduce oxidative stress, and improve skeletal muscle oxidative capacity (136). However, human trials to date have yielded mixed results, with improvements in tissue NAD^+^ content and selected mitochondrial markers but limited or no effect on whole-body insulin sensitivity in otherwise healthy or insulin-resistant adults (117). Mitochondria-targeted antioxidants such as MitoQ and the tetrapeptide SS-31 (elamipretide) can attenuate mitochondrial reactive oxygen species and improve endothelial or organ-specific function in experimental and early clinical settings, but consistent benefits on skeletal muscle performance and metabolic outcomes remain to be demonstrated (137–139). Together, these data suggest that mitochondrial-focused nutritional approaches may help counteract lipid accumulation and oxidative stress in skeletal muscle, but their role in T1D-associated myosteatosis is still largely inferential and requires targeted mechanistic trials.

The exercise and nutritional evidence in T1D varies considerably in strength. Trials of structured exercise have generally been short, modestly powered, and have used glycemic and fitness endpoints rather than muscle composition, so effects on intramuscular lipid are largely inferred. Nutritional evidence is more limited still, with leucine and omega-3 studies small and directed at protein turnover or nerve outcomes rather than myosteatosis. No intervention trial to date has used muscle fat fraction as a primary endpoint in T1D.

### 5.3 Pharmacological strategies

The pharmacological targets considered here map directly onto the cascade: myostatin to the myokine step, AMPK and PPAR agonists to nutrient sensing, and SIRT1 activators to mitochondrial quality control. Although no pharmacotherapies are currently approved specifically for T1D-associated myosteatosis, several mechanistically relevant agents have shown potential to modulate muscle lipid turnover, mitochondrial function, and inflammation. Among these, inhibition of the myostatin/activin pathway has generated considerable interest. Myostatin neutralization with monoclonal antibodies or receptor blockers increases lean mass, enhances oxidative metabolism, and reduces intramyocellular lipid content in metabolic disease models (76, 77). In humans, the activin receptor blocker bimagrumab increased skeletal muscle mass and markedly reduced total and visceral adipose tissue in individuals with T2D and obesity, indicating potent effects on lipid redistribution and mitochondrial function that may be relevant for T1D (140). Follistatin-based therapies show similar anabolic and lipolytic effects in early-phase studies, though data in autoimmune diabetes remain limited (105).

Agents that enhance cellular energy sensing represent another therapeutic avenue. Pharmacologic AMPK activation, via AICAR derivatives or next-generation AMPK agonists, stimulates fatty-acid oxidation, mitochondrial biogenesis, and autophagy, while suppressing pro-inflammatory signaling via NF-κB and NLRP3 pathways (48). Preclinical studies demonstrate that AMPK activation reverses lipid-induced mitochondrial stress and improves insulin action in skeletal muscle, though translation to T1D has been constrained by concerns over glycemic variability (141). Similarly, SIRT1 activators such as resveratrol and SRT2104 promote mitochondrial quality control and redox homeostasis via PGC-1α activation and NF-κB suppression. In small human trials, resveratrol improved insulin sensitivity and reduced markers of oxidative stress, although effects on muscle lipid composition and mitochondrial function require further validation (142, 143).

**Figure 4:**
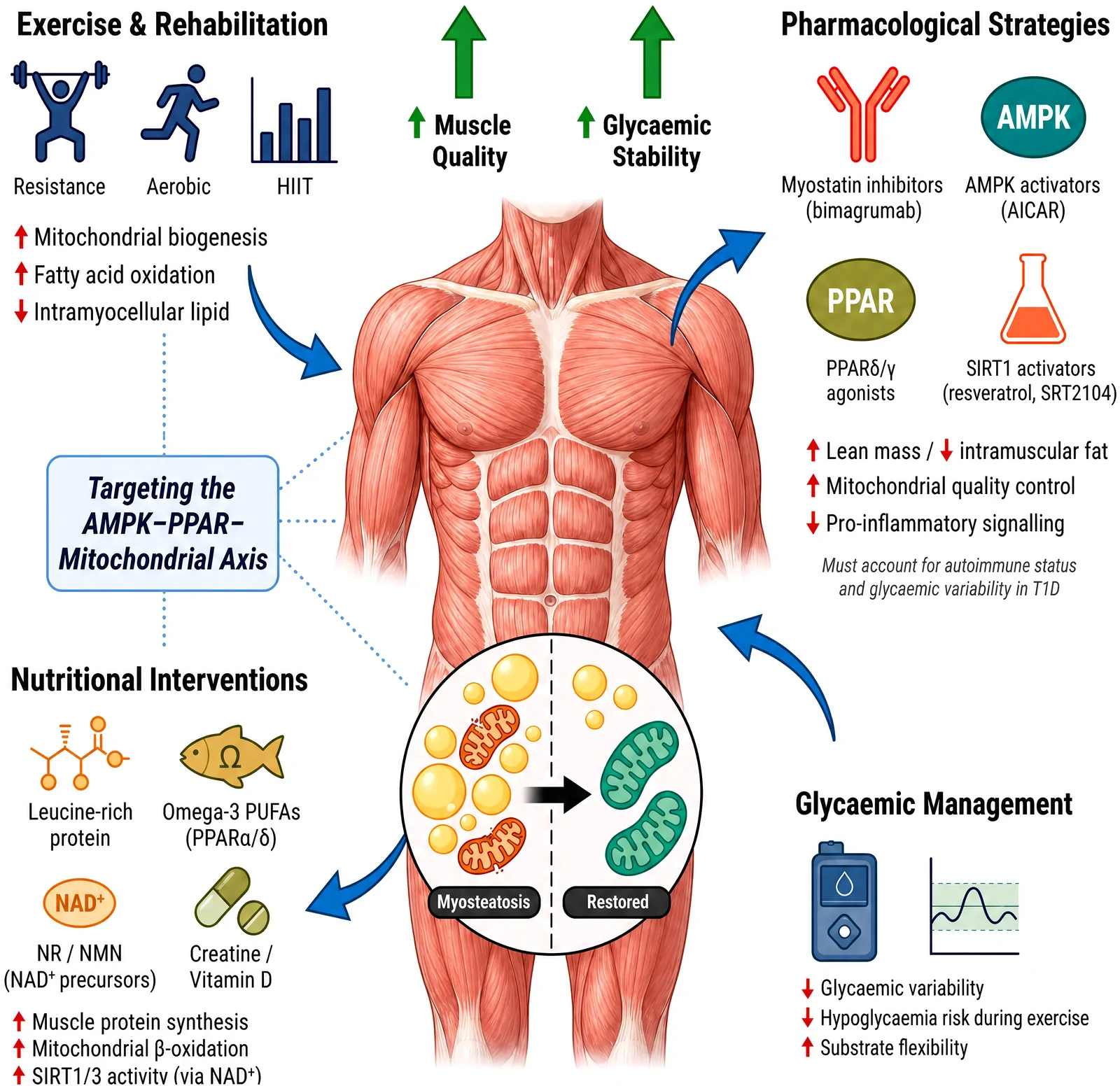
Multimodal therapeutic strategies to improve muscle quality and glycemic stability in T1D-associated myosteatosis. Radial schematic depicting four complementary intervention domains (Exercise and Rehabilitation, Nutritional Interventions, Pharmacological Strategies, and Glycemic Management) converging via bold blue arrows onto a central anatomical torso with fully rendered skeletal musculature, illustrating the integrated therapeutic framework required to address myosteatosis in T1D. A circular inset at the lower center contrasts the myosteatotic state (left half: gold lipid droplets crowding dysfunctional mitochondria) with the restored state (right half: reduced lipid burden and healthy teal-green mitochondria), anchoring the overarching therapeutic goals of improved muscle quality and glycemic stability (bold green upward arrows, upper center). All four domains converge mechanistically on the AMPK-PPAR-mitochondrial axis (central dashed-border box), reflecting the shared molecular target underlying each intervention class. Exercise and Rehabilitation (upper left) encompasses resistance training, aerobic exercise, and HIIT, each represented by corresponding activity icons; the three key metabolic outcomes are improved mitochondrial biogenesis, enhanced fatty-acid oxidation, and reduced intramyocellular lipid deposition. Nutritional Interventions (lower left) include leucine-rich protein to support muscle protein synthesis, omega-3 PUFAs acting via PPARα/δ activation to promote lipid clearance and dampen NF-κB-driven inflammation, NAD^+^ precursors (NR and NMN) to restore SIRT1/3-dependent mitochondrial quality control, and creatine with vitamin D to augment GLUT4 translocation and reduce inflammatory burden; collectively these enhance mitochondrial β-oxidation and SIRT1/3 activity. Pharmacological Strategies (upper right) under investigation include myostatin pathway inhibitors such as bimagrumab (Y-shaped antibody icon, coral), AMPK activators such as AICAR (teal oval), PPARδ/γ agonists (olive oval), and SIRT1 activators including resveratrol and SRT2104 (flask icon), targeting lean mass preservation, mitochondrial quality control, and suppression of pro-inflammatory signaling; a cautionary note indicates that all pharmacological approaches must account for autoimmune status and glycemic variability specific to T1D. Glycemic Management (lower right) encompasses continuous glucose monitoring, automated insulin delivery, and time-in-range optimization, acting to reduce glycemic variability and hypoglycemia risk during exercise, thereby enabling sustained engagement with lifestyle therapy and attenuating the metabolic stress that drives myosteatotic remodeling. Red ↑ and ↓ symbols denote therapeutic increases and decreases relative to the untreated myosteatotic state. AICAR, 5-aminoimidazole-4-carboxamide ribonucleotide; AMPK, AMP-activated protein kinase; CGM, continuous glucose monitoring; GLUT4, glucose transporter 4; HIIT, high-intensity interval training; NAD^+^, nicotinamide adenine dinucleotide; NF-κB, nuclear factor kappa B; NMN, nicotinamide mononucleotide; NR, nicotinamide riboside; PPAR, peroxisome proliferator-activated receptor; PUFA, polyunsaturated fatty-acid; SIRT1/3, sirtuin 1/3; SRT2104, selective sirtuin 1 activator; T1D, type 1 diabetes.

Modulators of nuclear receptor signaling, particularly PPAR agonists, offer additional potential for targeting myosteatosis. PPARδ agonists enhance skeletal muscle fatty-acid oxidation, shift fiber composition toward a more oxidative phenotype, and improve exercise tolerance in both animal models and early-phase human studies (144). Importantly, PPARδ activation promotes macrophage polarization toward an M2-like anti-inflammatory phenotype, thereby attenuating the lipid-driven inflammation that contributes to muscle metabolic dysfunction. PPARγ agonists, while beneficial for insulin sensitivity, carry risks of weight gain and oedema, making them less suitable for long-term use in T1D; nonetheless, selective PPAR modulators with reduced off-target effects are under investigation (144, 145). Despite the promise of these pathways, pharmacologic interventions in T1D must be evaluated with careful attention to autoimmune status, glycemic variability, and potential effects on β-cell or immune function. Many metabolic agents exert immunomodulatory actions beneficial in some contexts, but potentially disruptive in autoimmune diabetes. It is important to emphasize that none of the pharmacological strategies discussed above have been evaluated in T1D-specific clinical trials targeting myosteatosis; their application to T1D must therefore be considered exploratory and hypothesis-generating rather than evidence-based at this stage. As such, future therapeutic frameworks will likely integrate pharmacological approaches with structured exercise, nutritional interventions, and advanced glucose-monitoring technologies to optimize safety and achieve durable improvements in skeletal muscle metabolic health (**Figure 4**).

None of the pharmacological strategies discussed has been tested in T1D for a muscle endpoint. AMPK activators and PPAR agonists remain preclinical for this indication, and the bimagrumab, resveratrol, and nicotinamide riboside data derive from populations with T2D or obesity. Translation faces specific obstacles in T1D: agents affecting substrate utilization may alter hypoglycemia risk in insulin-treated individuals, and PPAR agonists carry established cardiovascular and skeletal safety considerations. These strategies are therefore presented as mechanistic rationale rather than as candidates ready for trial.

## 6 Synthesis and future perspectives

Myosteatosis in T1D is increasingly recognized as a distinct metabolic and inflammatory phenotype arising from the intersection of immune dysregulation, altered lipid handling, and mitochondrial dysfunction. Unlike classical muscle atrophy, myosteatosis reflects pathological lipid deposition within skeletal muscle fibers, driven by chronic low-grade inflammation, impaired substrate selection, and redox imbalance. The unique metabolic backdrop of T1D characterized by early-life insulin deficiency, glycemic variability, and lifelong autoimmune activation creates a permissive environment for premature declines in muscle quality and oxidative capacity.

This review sets out a model built on two feedback loops: one between mitochondrial damage and innate immune activation, and one between lipotoxic intermediates and substrate selection. Both are entered from the T1D-specific initiating conditions of insulin deficiency, glycemic variability, and sustained autoimmune activation, and both converge on progressive intramyocellular lipid accumulation without a requirement for obesity. Understanding these bidirectional interactions is essential for defining disease mechanisms and developing targeted interventions. Rapid advances in molecular imaging, multi-omics profiling, and high-throughput biomarker discovery offer unprecedented opportunities to detect and quantify myosteatosis with greater precision. These platforms enable delineation of lipid species, mitochondrial states, immune signatures, and their temporal dynamics across disease stages. Integrating such datasets with clinical phenotypes including fitness metrics, glycemic variability, and complication trajectories will be critical for establishing myosteatosis as a clinically meaningful endpoint in T1D.

### 6.1 Limitations and evidence gaps

Several important limitations warrant acknowledgment. The evidence supporting each step of the cascade proposed in this review varies considerably in strength, and **Table 2** sets this out explicitly, classifying each step as demonstrated, partial, inferred, or untested in T1D. Of the twelve steps, five are directly demonstrated in T1D, three are partially supported, three are inferred from obesity or T2D, and one has not been tested in this population. This distinction is material to how the model should be read: the bioenergetic steps that anchor the cascade rest on human T1D evidence, whereas the lipotoxic and immune-cell steps that follow it are extrapolated. One limitation cuts across all of them: the majority of mechanistic evidence cited here derives from studies of T2D, obesity, and aging rather than T1D specifically, and direct human data using gold-standard techniques such as ¹H-MRS and muscle biopsy remain limited to a small number of cross-sectional studies with modest sample sizes (17, 21, 34, 35). The specific limitations of the evidence in each domain are stated at the close of Sections 2.3, 2.4, 4.2, 4.3, 5.2, and 5.3 rather than consolidated here. Together these gaps define the need for longitudinal, mechanistic, T1D-specific studies to test the framework proposed in this review.

**Table 2:** Evidence status in type 1 diabetes for each step of the proposed mechanistic cascade.

Step in cascade	Evidence status in T1D	T1D-specific sources
Insulin deficiency and peripheral hyperinsulinemia impair muscle glucose disposal	Demonstrated	(6, 7, 12)
Glycemic variability induces oxidative and inflammatory stress	Demonstrated	(5, 13)
Chronic low-grade systemic inflammation independent of adiposity	Demonstrated	(13, 14)
Mitochondrial dysfunction and impaired oxidative capacity in skeletal muscle	Demonstrated	(17, 19, 21, 34, 35)
Impaired fatty-acid oxidation with accumulation of ceramides and diacylglycerols	Inferred	None; extrapolated from obesity and type 2 diabetes
Dysregulation of AMPK, mTORC1 and PPAR nutrient sensing in muscle	Inferred	None; not quantified in T1D muscle biopsy
Myokine dysregulation (myostatin, irisin, IL-15)	Partial	(61, 77, 81)
Macrophage and CD8^+^ T-cell infiltration of skeletal muscle	Inferred	None; not characterized by immunohistochemistry in T1D
Denervation as a contributor to intramuscular fat	Partial	(94, 98, 99, 101)
Exercise improves muscle oxidative metabolism and glycemic outcomes	Demonstrated	(123, 124, 127, 128)
Nutritional interventions modify muscle protein or nerve outcomes	Partial	(130–132)
Pharmacological targeting of the AMPK-PPAR axis	Not tested	None; no T1D trial

The gaps identified in **Table 2** map onto five specific studies. First, quantification of ceramide and diacylglycerol species in skeletal muscle biopsies from individuals with T1D, paired with intramyocellular lipid measurement, would establish whether the lipotoxic intermediates inferred from T2D are present in this population. Second, phosphoprotein quantification of AMPK, mTORC1, and PPARδ signaling in the same biopsy material would test the nutrient-sensing arm of the model, which has not been measured directly in T1D muscle. Third, immunohistochemical characterization of macrophage and CD8^+^ T-cell infiltration in an adequately powered T1D cohort would determine whether the immune-cell step occurs at all in this disease. Fourth, studies combining compartment-specific muscle fat quantification with electrophysiological assessment of motor unit integrity would separate the neurogenic and immunometabolic contributions to intramuscular fat. Fifth, longitudinal cohorts combining imaging, omics, and metabolic phenotyping would define the natural history of myosteatosis in T1D and identify which pathways are modifiable. Only after these have been addressed would a pharmacological trial targeting the AMPK-PPAR axis in T1D be justified.

## 7 Conclusion

Mechanistically informed interventions targeting immune-metabolic mitochondrial cross-talk hold particular promise. Precision approaches that incorporate structured exercise programs, tailored nutritional strategies, and pharmacological modulation of PPARδ, AMPK, SIRT1, and NAD^+^-dependent pathways represent candidate strategies once the mechanistic basis is established in T1D. Ultimately, addressing myosteatosis in T1D has the potential to improve not only skeletal muscle resilience but also systemic glucose homeostasis, cardiometabolic health, and long-term quality of life. Recognizing and treating this underappreciated component of T1D pathophysiology may represent an important step toward more comprehensive metabolic care in autoimmune diabetes.

## Glossary

H-MRS: Proton magnetic resonance spectroscopy
P-MRS: Phosphorus-31 magnetic resonance spectroscopy
4-HNE: 4-hydroxynonenal
AICAR: 5-aminoimidazole-4-carboxamide ribonucleotide
Akt: Protein kinase B
AMPK: AMP-activated protein kinase
AMPKα: AMP-activated protein kinase alpha subunit
ATP: Adenosine triphosphate
CD8: Cluster of differentiation 8
CGM: Continuous glucose monitoring
CRP: C-reactive protein
DAG: Diacylglycerol
DAMPs: Damage-associated molecular patterns
DXA: Dual-energy X-ray absorptiometry
EV: Extracellular vesicle
FNDC5: Fibronectin type III domain-containing protein 5
GLUT4: Glucose transporter 4
HbA1c: Hemoglobin A1c
HIIT: High-intensity interval training
IFN-γ: Interferon-gamma
IL-1β: Interleukin-1beta
IL-6: Interleukin-6
IL-15: Interleukin-15
IRS-1: Insulin receptor substrate-1
JAK: Janus kinase
JNK: Jun N-terminal kinase
MAPK: Mitogen-activated protein kinase
MDA: Malondialdehyde
M1: Classically activated macrophage phenotype
M2: Alternatively activated macrophage phenotype
MitoQ: Mitoquinone
MRI: Magnetic resonance imaging
MRS: Magnetic resonance spectroscopy
mtDNA: Mitochondrial DNA
mTOR: Mechanistic target of rapamycin
mTORC1: Mechanistic target of rapamycin complex 1
NAD⁺: Nicotinamide adenine dinucleotide
NF-κB: Nuclear factor kappa B
NK: Natural killer
NLRP3: NOD-like receptor family pyrin domain-containing 3
NMN: Nicotinamide mononucleotide
NR: Nicotinamide riboside
PGC-1α: Peroxisome proliferator-activated receptor gamma coactivator 1 alpha
PPAR: Peroxisome proliferator-activated receptor
PPARα: Peroxisome proliferator-activated receptor alpha
PPARδ: Peroxisome proliferator-activated receptor delta
PPARγ: Peroxisome proliferator-activated receptor gamma
PUFAs: Polyunsaturated fatty-acids
ROS: Reactive oxygen species
SAA: Serum amyloid A
SOCS3: Suppressor of cytokine signaling 3
SIRT1: Sirtuin 1
SIRT3: Sirtuin 3
SS-31: Elamipretide
SRT2104: Sirtuin 1 activator SRT2104
STAT3: Signal transducer and activator of transcription 3
TLR: Toll-like receptor
TNF-α: Tumor necrosis factor-alpha.
